# Effect of Humid Air Exposed to IR Radiation on Enzyme Activity

**DOI:** 10.3390/ijms23020601

**Published:** 2022-01-06

**Authors:** Olga I. Yablonskaya, Vladimir L. Voeikov, Kirill N. Novikov, Ekaterina V. Buravleva, Valeriy A. Menshov, Aleksei V. Trofimov

**Affiliations:** 1 Emanuel Institute of Biochemical Physics of Russian Academy of Sciences, 119334 Moscow, Russia; vinoman66@mail.ru (V.A.M.); avt_2003@mail.ru (A.V.T.); 2 Faculty of Biology, Lomonosov Moscow State University, 119234 Moscow, Russia; v109028v1@yandex.ru (V.L.V.); kirniknov@yandex.ru (K.N.N.); b_u_k_a@mail.ru (E.V.B.)

**Keywords:** IR radiation, horseradish peroxidase, catalase, alkaline phosphatase, antioxidant, vapor, coherent domain

## Abstract

Water vapor absorbs well in the infra-red region of the electromagnetic spectrum. Absorption of radiant energy by water or water droplets leads to formation of exclusion zone water that possesses peculiar physico-chemical properties. In the course of this study, normally functioning and damaged alkaline phosphatase, horseradish peroxidase and catalase were treated with humid air irradiated with infrared light with a wavelength in the range of 1270 nm and referred to as coherent humidity (CoHu). One-minute long treatment with CoHu helped to partially protect enzymes from heat inactivation, mixed function oxidation, and loss of activity due to partial unfolding. Authors suggest that a possible mechanism underlying the observed effects involves altering the physicochemical properties of aqueous media while treatment of the objects with CoHu where CoHu acts as an intermediary.

## 1. Introduction

In our recent research we the observed changes in physicochemical properties of aqueous systems after non-chemical treatment with humid air exposed to IR radiation that was called coherent humidity (CoHu) [1]. These changes were the redox potential, dielectric constant values alterations along with a change in buffer properties of water and buffers. This indicates of charge distribution alteration and the presence of heterogeneity in the treated aqueous medium, which is typical to exclusion zone (EZ) water [2,3]. Exclusion zone, formed on the interfaces of water and hydrophilic surfaces, excludes particles and solutes—the property to which it owes its name.

Proteins play a key role in the function of cells and tissues. Catalytic, transport, structural, protective, regulatory, and motor functions are the most common and global functions of proteins. Composed of hundreds of amino acids, proteins possess a complex, three-dimensional structure. Their constituent amino acid sequences are encoded in the genome, and the components of protein biosynthesis form one of the most impressive example of machinery in nature.

Protein oxidation is a well-known reason for aging [4]. As proteins are responsible for managing a vast number of cell functions, their oxidation leads to a gradual accumulation of faults and cellular failures. Proteins are oxidized by reactive oxygen species (ROS), such as hydrogen peroxide, singlet oxygen, organic peroxides, nitric oxide, and hydroxyl and superoxide radicals. These compounds are highly reactive and are capable of damaging both small and large organic molecules due to their possession of valent, unpaired electrons allowing for nucleophilic attack of nearby cellular components. An imbalance in ROS generation and its elimination leads to oxidative stress [5]. Oxidation of a protein leads to the formation of chemical radicals within amino acids themselves and increases the chance of structural deformation. 

Protein stability is one of the parameters that allow cells to withstand oxidative stress and premature aging. Cysteine residues are especially sensitive to oxidation because the thiol group in cysteine can be oxidized in several ways to form sulfenic acid (SOH), a disulfide bond (S-S), and sulfinic (SO_2_H) or sulfonic acids (SO_3_H). The latter two are formed irreversibly [6,7]. Thiol groups of cysteine residues are secured against oxidation and other external influences because they play an important role in the protected catalytic and regulatory sites within proteins and enzymes [8]. However, the structural rigidity of proteins lessens as they age, which may lead to the exposure of catalytic sites and thiol groups to oxidation and other damaging factors. 

There are chemical and physical ways to protect biological macromolecules from premature damage. Infrared light treatment as a physical influence has shown particularly interesting results. It has been demonstrated that in vivo treatment with near-infrared light at 670 nm reduced the negative effects of traumatic injury to the central nervous system in the form of oxidative stress in the optic nerve, secondary to a decrease in ROS production and metabolism and resulting in restored visual function [9,10]. Other research suggests that brief (2 min, 24 s) 670-nm LED treatments in the course of photobiomodulation may enhance recovery from retinal injury and other ocular diseases in which mitochondrial dysfunction is postulated to play a role [11]. Infrared LED (855 nm) irradiation resulted in a decrease in ROS production following the induction of oxidative stress in human dental pulp cells by lipopolysaccharide without a significant effect on cellular metabolism [12]. The use of a 950 nm LED in a pilot study that investigated the analgesic efficacy of light-emitting diode therapy suggested a general analgesic effect of LED irradiation in favor of the experimental group, although it was not statistically proven [13]. The same authors demonstrated enhanced wound healing in case of treatment with the red-light emitting diode [14]. Lee S.Y.C. et al. concluded that 1072 nm infrared light had a photobiomodulation effect which resulted in an enhanced immune response to cutaneous bacterial infection in mice and significantly increased expression of key immunomodulatory genes [15]. In another study, irradiation with a 904 nm low-level laser reduced inflammatory cell migration in mice with lypopolysaccharide-induced peritonitis in a dose-dependent manner [16].

Danno et al. found that IR radiation suppresses UV-B induced sunburn [17]. Menezes and coworkers have suggested that preliminary irradiation with IR light, which happens during sunrise, is a natural mechanism of cell protection against the solar UV radiation, and have shown that visible to near IR radiation at 400–2000 nm protects human dermal fibroblasts from UV-A and UV-B cytotoxicity [18]. 

Interestingly, IR also increases the width of exclusion zones (EZ) in water that emerge at natural and artificial hydrophilic surfaces spontaneously [19,20]. It is quite possible that “base-line” environmental IR radiation fuels the formation of EZ [21]. Thus, all cellular membranes and surfaces of macromolecules can be considered as hydrophilic interfaces where EZ originates in living organisms that consist mostly of water. 

Since the mid-20th century, it has been postulated that the protein folding process, which is essential for establishing a protein’s function, is caused by an opposite shift in entropy between polypeptide and solvent [22,23]. In protein folding conformational entropy loss occurs along with translational entropy gain in the solvent. Protein molecules fold under the influence of thermal pressure, which differs at the protein surface and in the bulk solvent. The proper natural tertiary and quaternary structures are obtained and maintained at maximum translational entropy in the surrounding aqueous media. The loss of conformational entropy consequent of protein folding is compensated for by the increase in hydration entropy, which results from the ‘freeing’ of water previously held in order by the hydrophobic residues at the aqueous interface, ultimately allowing the protein to fold.

In this study, we have proceeded to investigate the ability of humid air illuminated with IR affect the activity of enzymes in aqueous medium that were exposed to damaging factors (heat, oxidation, and chemical denaturants). 

## 2. Results

### 2.1. Establishing Enzymatic Activity Controls

**Figure 1:**
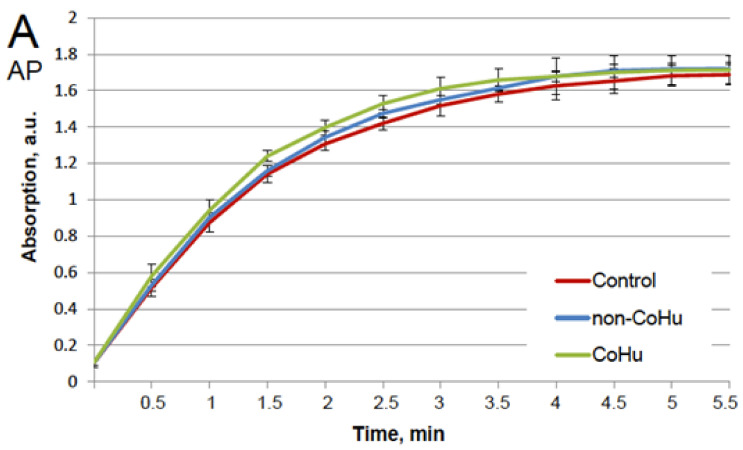

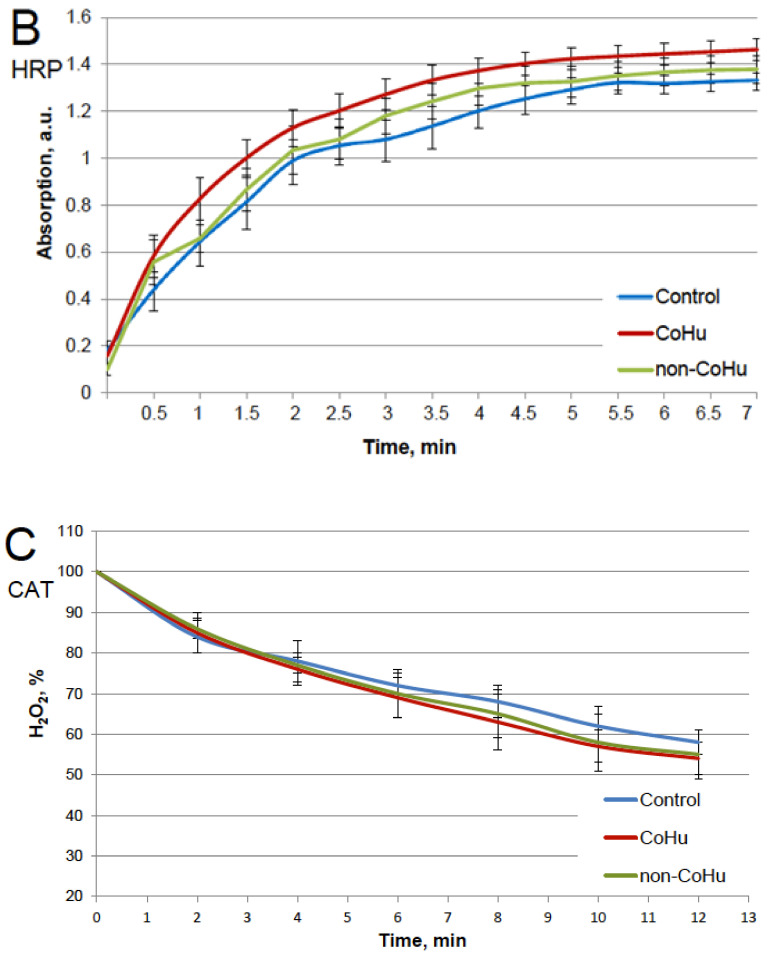
Effect of CoHu on alkaline phosphatase ((**A**), AP), HRP (**B**) and catalase ((**C**), CAT) activity in vitro. Data presented as mean arbitrary units of 7–8 measurements ± SD. Control—the activity of enzymes samples that were not treated with humid airstram. CoHu—the activity of enzymes that were treated with CoHu, and non-CoHu—the activity in control samples.

Our first task was to find out whether CoHu has any effect upon normally functioning (non-inactivated) enzymes. For that purpose, all enzyme samples were divided in 3 groups as shown in the legend. The data presented in Figure 1A show the effect of CoHu on normally functioning alkaline phosphatase, Figure 1B—horseradish peroxidase and Figure 1C—the effect of CoHu on intact catalase. For catalase (C), residual quantity of H_2_O_2_ (catalase substrate) in the catalase-assisted degradation reaction is shown, which reflects the activity of the enzyme. The figure shows that CoHu does not have any significant impact on undamaged enzyme activity. Data from this figure will be used as intact enzyme control in other experiments. 

### 2.2. Heating Inactivation Test 

**Figure 2:**
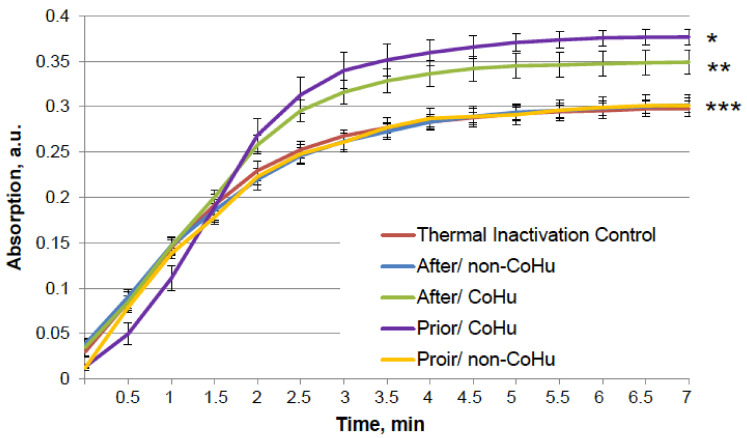
The effect of CoHu on thermally inactivated HRP. Data presented as mean arbitrary units of 8 measurements ± SD. Prior/non-CoHu means that samples were treated with non-CoHu before thermal inactivation. After/CoHu means that samples were treated with CoHu mode after thermal inactivation. Prior/CoHu means that samples were treated with CoHu before thermal inactivation. *, ** and ***—Lines with different superscripts differ significantly (*p* < 0.05).

The results for HRP are presented in Figure 2. After heating the activity of HRP decreased 4.5 times compared to intact enzyme, the mean of 0.308 a.u. against 1.360 a.u (Figure 1B). Samples treated with CoHu before and after inactivation had higher activity than the rest of the samples. Moreover, treatment of the enzyme before inactivation had a stronger effect than after, which indicates a possible protective effect. The mean increase in peroxidase activity in the end of the reaction in samples treated with CoHu before and after inactivation was 24.9% and 14.9%, respectively, compared to inactivated control. In Figure 2 the *p* value between the end points marked with * and *** is 0.0287, between * and ** *p* is 0.0485, and between ** and *** *p* is 0.0366.

**Figure 3:**
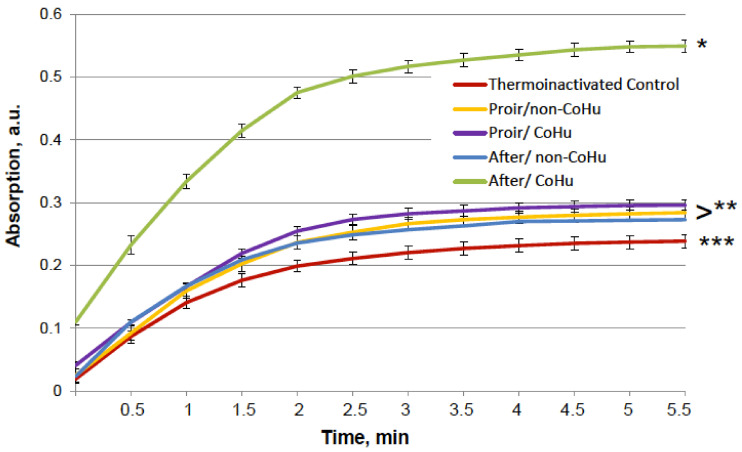
Effect of CoHu on thermally inactivated alkaline phosphatase activity. Data presented as mean arbitrary units ± SD of 10 measurements. Prior/non-CoHu means that samples were treated with non-CoHu before thermal inactivation. After/CoHu means that samples were treated with CoHu after thermal inactivation. Prior/CoHu means that samples were treated with CoHu before thermal inactivation. *, ** and ***—Lines with different superscripts differ significantly (*p* < 0.05).

The same type of test was performed with alkaline phosphatase. Heating of alkaline phosphatase resulted in a 7-fold loss of its activity. However, Figure 3 shows that treatment of inactivated enzyme samples with CoHu after inactivation had the most remarkable restoring effect. The activity of the enzyme in all other samples that were treated with CoHu was also higher than in heat-inactivated controls that had only 14% of the activity of intact enzyme (ref. Figure 1A). The mean increase in alkaline phosphatase activity in the end of the reaction in samples treated with CoHu after inactivation was 130% in the last point of the measurement compared to inactivated control, which was 32% of the activity of intact enzyme. Optical density of the product of the reaction in samples that were treated with CoHu before heating differs from inactivated control (*p* = 0.032). However, there is no significant difference between samples treated with non-CoHu.

**Figure 4:**
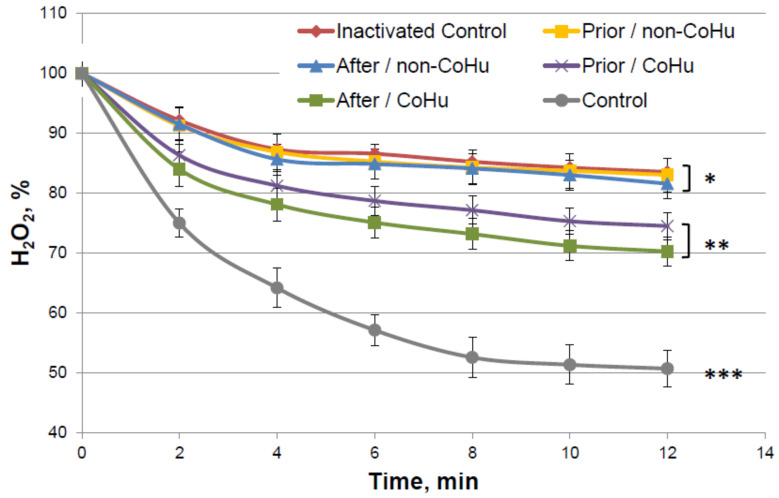
Residual quantity of H_2_O_2_ (catalase substrate) in the catalase-assisted degradation reaction. Data reflect the activity of the enzyme. *, ** and ***—Lines with different superscripts differ significantly.

Figure 4 shows the dynamics of changes in the content of hydrogen peroxide during an enzymatic reaction. It was shown that in thermally inactivated controls the substrate is not much consumed (83.5% of H_2_O_2_ remains versus 57% of substrate in the reaction with intact enzyme), but in the samples treated with CoHu, the substrate consumption is increased, therefore, the enzyme activity is increased. Activity comparison was based on the data of the last points of the plots. Treatment with CoHu before or after thermal inactivation allowed to partially restore the catalytic functions of the enzyme (+17.8% and +13.4%, respectively). There is no statistically significant difference between data of these two groups of samples (*t*-test, *p* > 0.05), however there are reliable differences between these two groups together, control and samples that were treated with non-CoHu. 

### 2.3. Oxidative Inactivation Test

**Figure 5:**
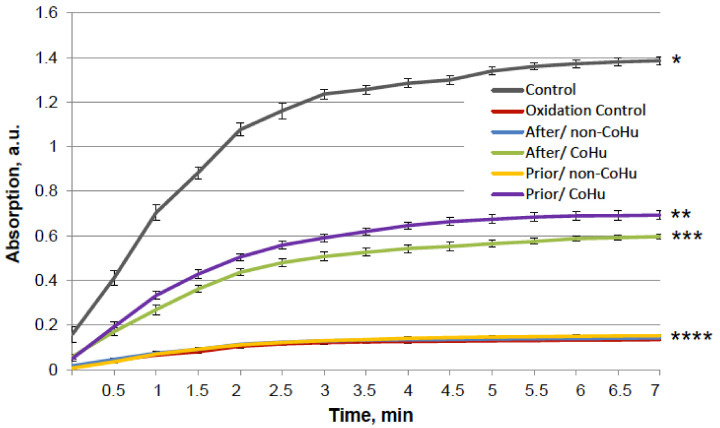
The effect of CoHu on oxidized HRP. Data presented as mean arbitrary units of 10 measurements ± SD. Prior/non-CoHu means that samples were treated with non-CoHu before inactivation. After/CoHu means that samples were treated with CoHu after inactivation. Prior/CoHu means that samples were treated with CoHu before inactivation. *, **, *** and ****—Lines with different superscripts differ significantly (*p* < 0.05).

Figure 5 shows that mixed function oxidation (MFO) decreased HRP activity 9.8 times. Treatment with non-CoHu did not cause changes in activity compared to oxidized control. The activity of samples treated with CoHu before oxidation made it possible to save the mean of 45.3% of HRP activity (450% of oxidized control), while samples treated after inactivation restored 42.4% (421% of oxidized control). In Figure 5 the *p* value between end points marked with * and **** is 0.0087, between end points marked with ** and **** *p* is 0.0479, and between ** and *** *p* is 0.0106. Thus, the enzymatic activity of HRP was either partially protected or restored by treatment of the samples with CoHu both prior to and after oxidation. The pH values were controlled in the samples before measurements.

**Figure 6:**
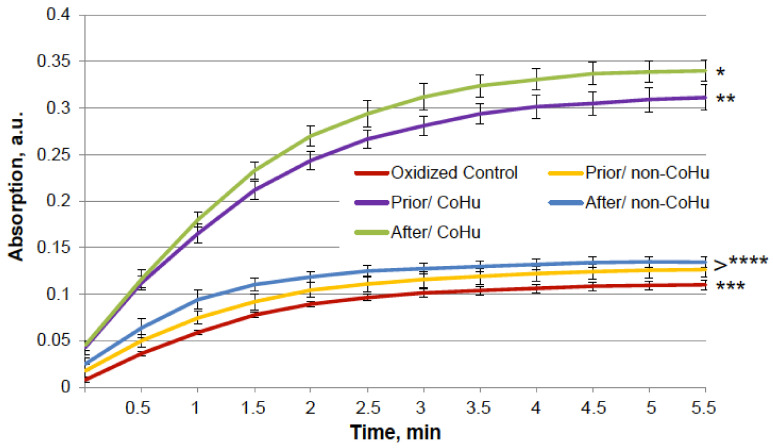
Effect of CoHu on oxidized alkaline phosphatase activity. Data presented as mean arbitrary units ± SD of 10 measurements. Prior/non-CoHu means that samples were treated with non-CoHu before inactivation. After/CoHu means that samples were treated with CoHu after inactivation. Prior/CoHu means that samples were treated with CoHu before inactivation. *, **, *** and ****—Lines with different superscripts differ significantly (*p* < 0.05).

In Figure 6, it is shown that treatment with CoHu before and after oxidation had a notable protective effect on the activity of alkaline phosphatase. Only 6.4% of alkaline phosphatase activity remained after oxidation in oxidized control samples (red line). Treatment with non-CoHu led to a slight increase of activity. Treatment with CoHu after inactivation helped to restore 19.8% of the intact enzyme activity (309% of oxidized control), while treatment before oxidation partially protected the enzyme and saved a mean of 18.1% of intact enzyme activity (282% of oxidized control). In Figure 6 the *p* value between lines marked with * and ** is 0.0481, between lines marked with ** and **** *p* is 0.0187, and between lines marked with *** and **** *p* is 0.0494. In Figure 6 the activity of the intact enzyme control is not shown to pay more attention to the details in the activity of inactivated samples. The control activity values can be found in Figure 1A. 

**Figure 7:**
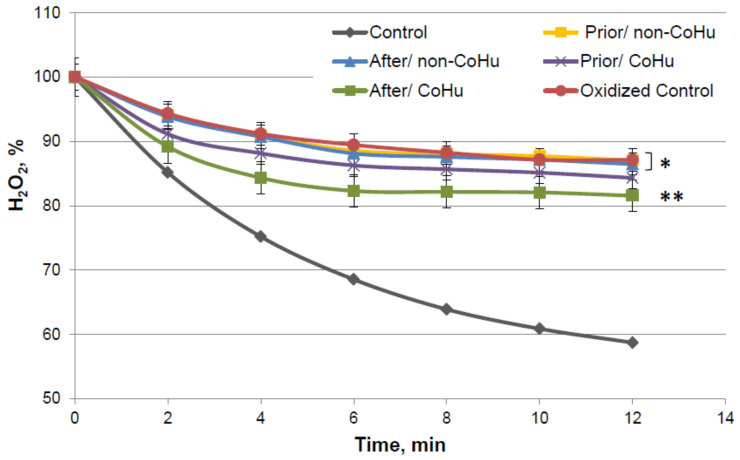
Residual quantity of H_2_O_2_ (catalase substrate) in the catalase-assisted degradation reaction. Data reflect the activity of the enzyme. * and **—Lines with different superscripts differ significantly (*p* < 0.05).

Figure 7 presents data of the MFO test with catalase. In this figure substrate consumption of intact enzyme is presented (grey line). Decrease of hydrogen peroxide concentration is dependent on the activity of the enzyme. The higher the hydrogen peroxide concentration in the end of the reaction—the lower the activity of the enzyme. 

By the 12-th minute of the measurement a mean of 59% of H_2_O_2_ remains in the medium of the undamaged enzyme, while oxidized catalase leaves 87.12% of the substrate unconsumed. Samples that were treated with non-CoHu did not differ from oxidized controls. A reliably increased consumption of H_2_O_2_ was observed in samples that were treated with CoHu after oxidation (81.55%, +5.57% to oxidized control). Samples treated with CoHu after inactivation are reliably different from the samples treated with non-CoHu (*p* = 0.0455). Treatment before oxidation tends to preserve the enzyme but not significantly (*p* = 0.068). Samples treated with CoHu before inactivation (purple line) are not reliably different either from the samples treated with non-CoHu or samples treated with CoHu after inactivation (green line).

### 2.4. Chemical Denaturant Test

Testing whether treatment with humidified air irradiated by IR could partially restore the activity of denatured enzymes was the next objective of this study. 

**Figure 8:**
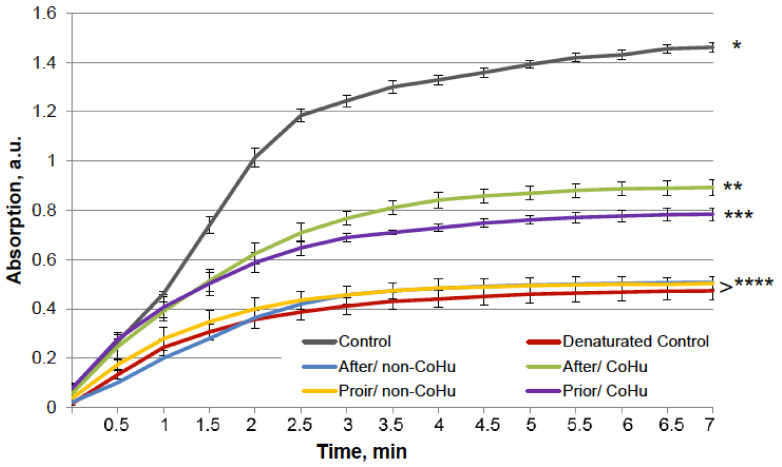
The effect of CoHu on denatured HRP. Data presented as mean arbitrary units ± SD of 8 measurements. Prior/non-CoHu means that samples were treated with non-CoHu before inactivation. After/CoHu means that samples were treated with CoHu after inactivation. Prior/CoHu means that samples were treated with CoHu before inactivation. *, **, *** and ****—Lines with different superscripts differ significantly (*p* < 0.05).

**Figure 9:**
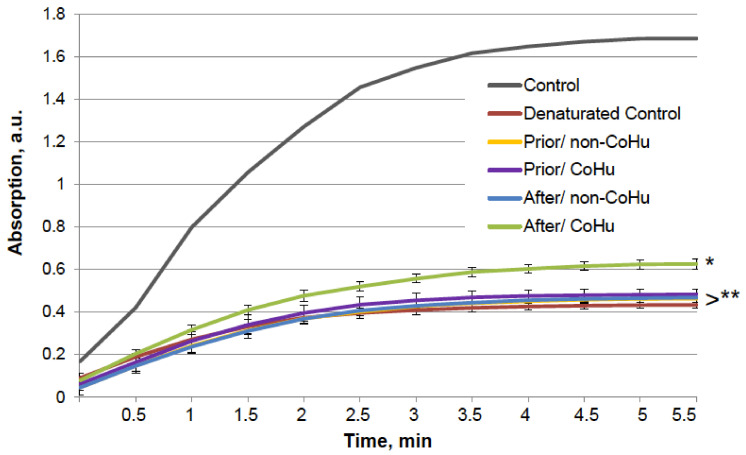
Effect of CoHu on denatured alkaline phosphatase activity. Data presented as mean arbitrary units ± SD. Control—activity of an intact enzyme not exposed to unfolding buffer. Prior/non-CoHu—activity in samples that were treated with non-CoHu before inactivation. After/CoHu—activity in samples that were treated with CoHu after inactivation. Prior/CoHu—activity in samples that were treated with CoHu before inactivation. * and **—Lines with different superscripts differ significantly (*p* < 0.05).

The results for HRP are presented in Figure 8. The grey line represents normal HRP activity as a control. After the unfolding, 32.2% of HRP activity remained (red line). Treatment with non-CoHu did not differ from denatured controls. A restoring effect was observed in response to the use of CoHu both before (53.4% of intact enzyme activity and 166% of denatured control) and after unfolding (60.9% of intact enzyme activity and 189% of denatured control). Therefore, treatment after denaturation had a greater impact. In Figure 9 the *p* value between lines marked with ** and *** is 0.0297, between lines marked with *** and **** *p* is 0.0213, and between lines marked with * and ** *p* is 0.0076.

A similar test was performed with alkaline phosphatase. The results are presented in Figure 9. The six groups of samples are similar to those in the previous experiment. The grey line shows the activity of a portion of normally functioning enzyme. Exposure to the unfolding solution decreased enzymatic activity to 23.9% in the unfolding control (red line). In the case of alkaline phosphatase, treatment with CoHu after unfolding allowed to partially restore the enzyme activity (35.9% of the intact enzyme activity and 150% to the unfolded control activity), while all other test set-ups showed no significant effect. 

**Figure 10:**
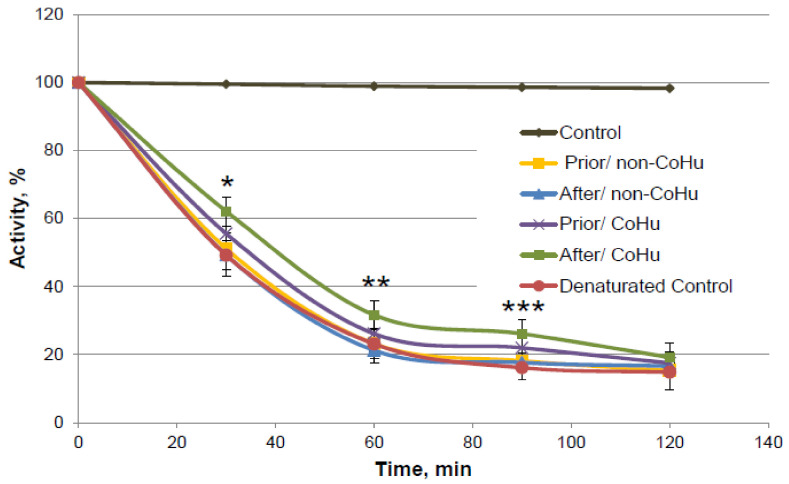
Catalase activity based on the residual quantity of H_2_O_2_ (catalase substrate) in the catalase-assisted degradation reaction. Control—activity of intact enzyme. Prior/non-CoHu—activity in samples that were treated with non-CoHu before inactivation. After/CoHu—activity in samples that were treated with CoHu after inactivation. Prior/CoHu—activity in samples that were treated with CoHu before inactivation. In time points marked as *, ** and *** there is reliable difference between the groups (*p* < 0.05).

As it is shown on Figure 10, there is a reliably significant effect of treatment with CoHu after denaturation compared to denatured control and samples that were treated with non-CoHu at 30 min, 60 min and 90 min. In time points marked as *, ** and *** there is reliable difference between the following three groups of samples: samples treated with CoHu after exposure to 2-ME, inactivated with 2-ME controls, and samples treated with non-CoHu (*p* < 0.05). At the last point at 120 min there is no significant difference between all the sample groups. 

## 3. Discussion

This pioneering study touches on several little-studied areas at once. First, the exposure of biological objects to light with a wavelength of around 1270 nm has been a rare study object, although the search for biological effects of irradiation with light in the infrared region has long been successful. Second, the effect of such irradiated humidity (CoHu) on the redox abilities of aquatic environments and enzymes, which occupy the basal, molecular level of life organization, has not been previously studied. In other studies, such as those mentioned in the introduction, the objects of observation were cells and tissues, leaving it unclear what molecular substances were responsible for the observed effects. An important detail of this study is that the objects observed herein were not irradiated directly but rather treated with irradiated humidified air (CoHu).

The test results show IR-treated humid airstream does not affect normally functioning enzymes (Figure 1). Treatment of the samples with the non-CoHu airstream did not affect the activity of thermally inactivated peroxidase and catalase. However, treatment of the thermally inactivated samples with CoHu both before and after inactivation significantly increased HRP and catalase activity (Figure 2 and Figure 4). As for alkaline phosphatase, exposure to non-CoHu slightly increased enzyme activity due to the additional air supply. The activity in the samples that were treated with CoHu before inactivation is comparable to the results with peroxidase. It is noteworthy that treatment with CoHu after inactivation markedly increased the activity of alkaline phosphatase (Figure 3).

In tests using mixed function oxidation (MFO), HRP and alkaline phosphatase activity was partially restored when the samples were treated with CoHu, both before and after inactivation (Figure 5 and Figure 6). The enzymes responded slightly differently to treatment with the irradiated humid airstream, but the general trend in enzyme activity is the same for both. Oxidized HRP activity was higher in the case of treatment with CoHu before oxidation, while oxidized alkaline phosphatase activity was higher in case of treatment after oxidation. Oxidized catalase activity was partially restored by treatment with CoHu after inactivation (Figure 7). Treatment with non-CoHu did not reveal any protective or restoring effect in tests with HRP and catalase (Figure 5 and Figure 7).

The chosen enzymes represent different classes with varying action mechanisms and substrates. Therefore, the damaging factors as heat, oxidation and denaturation affect particular molecule parts in every case. In some circumstances damaging effects can combine. Thus, at the base of MFO systems action a 3-step modified metal-catalyzed Haber-Weiss cycle is proposed [24,25]:

ascorbate + 2M^n+^   →   2M^(n−1)+^ + dehydroascorbate + 2H^+^ (1)

ascorbate + O_2_   →   dehydroascorbate + H_2_O_2_ (2)

M^(n−1)+^ + H_2_O_2_   →   M^n+^ + OH^•^ + OH^−^ (3)

In this context, reduced metals and hydrogen peroxide interact to form a highly reactive OH^•^ by a Fenton mechanism. Meanwhile, Cu^2+^ is capable to binding to HRP, leading to a conformational change, causing partial denaturation of HRP to an extent of 54% [26]. Thus, the HRP activity decrease was partially due to oxidation and denaturation combined. Inactivation of HRP by excess amounts of H_2_O_2_ by mechanism-based pathway has been well described [27,28]. Reversible and irreversible mechanisms of HRP inactivation have been proposed. At H_2_O_2_ concentrations below 1.0 mM, a reversible mechanism prevails, while at higher H_2_O_2_ concentrations, irreversible inactivation takes place. This is a plausible explanation for the observed HRP inactivation as hydrogen peroxide is formed in MFO. The regenerative and protective effect of CoHu on HRP under these conditions, therefore, should be associated with its antioxidant properties. 

Catalase activity was partially restored when samples were treated with CoHu after inactivation, while treatment before inactivation had a tendency to protect the enzyme (Figure 10). Copper affects the secondary structure when it binds near a heme group in catalase, as well as the binding of Cu^2+^ to bovine liver catalase destroyed H-bonds [29]. We specifically used the least damaging effect and the lowest effective concentration of the MFO so that irreversible damage to the enzymes did not occur, after which there would be no hope of any restoration of function. Treatment of oxidized samples with air humidity irradiated with infrared light has shown good protective and reducing properties with respect to oxidation. The question remains whether this effect can be translated to an entire organism in vivo.

The employed unfolding buffer affected the thiol bonds in proteins. When HRP, alkaline phosphatase and catalase structures were disturbed in the unfolding buffer, the activities of the enzymes were increased by treatment of the samples with CoHu after inactivation (Figure 8, Figure 9 and Figure 10). HRP was partially preserved even when it was treated with air humidity before exposure to unfolding (Figure 8). 

The wavelength range around 1270 nm was not chosen randomly. The idea was to study the effects of a wavelength close to the one of singlet oxygen phosphorescence [24]. To date, many claims have been made about the positive role of reactive oxygen species (ROS) in cell signaling, gene expression [25], cell differentiation, apoptosis, and immune and nervous system functioning [30]. 

Singlet oxygen, with the oxygen atom in the singlet ^1^∆_g_ state, occupies a special place among ROS [31]. This short-living ROS can be detected by its emission at 1268 nm during O_2_(^1^Δ_g_) → O_2_ (^3^Σ^−^_g_) transition in enzymatic reactions and in cells [32]. Singlet oxygen is characterized by a short maximum lifetime of 0.6 µs [33] and, therefore, can only travel short distances, roughly 100 nm, from its place of origin [34]. It has a relatively short list of direct reactions, yet its contribution to cell signaling and oxidative stress correction is crucial. Singlet oxygen is a major agent generated by many different cell types, especially by neutrophils in the course of their immune response to infection. Interestingly, water absorbs intensely in the IR region of 1270 nm [35]. 

Krasnovskiy and his group have shown that irradiation of water and organic solvents saturated with air at a wavelength of 1268 nm with the power of 30–150 mW led to formation of singlet oxygen [36]. However, the concentration of free oxygen is significantly lower in tissues, which reduces the rate of O_2_(^1^Δ_g_) formation.

Later Anquez et al. showed that the 1270 nm direct photosensitizer-free laser irradiation initiated singlet oxygen formation in cell culture and induced cancer cell death [37,38]. The lethal effect depended on the radiation wavelength, intensity and oxygen content in the media. However, this publication raised doubts that the observed cell death was due to photoexcitation of dissolved oxygen as the primary absorber of 1270 nm light in the biological environment is water due to the low extinction coefficient for molecular oxygen at 1270 nm [39]. Another argument to support this suggestion is that biological effects appear not only at irradiation at 1270 nm exactly, but at the range of about 1200–1300 nm, where water absorbs intensely.

Water plays a crucial role in protein folding, action, and dynamics [40]. From the thermodynamic point of view, the balance between intramolecular forces and hydration energy of water keeps protein structure stable and facilitates conformational changes [41,42]. Data from crystallography, neutron scattering, and simulations allowed proposing that water layer at protein molecule surface is 10% more dense than bulk water [43,44] and it has a reduced freezing point [45]. The content of such non-freezing water was 9–15% in coacervating systems (lactoferrin/ß-lactoglobulin (heteroprotein) coacervate and others). There is evidence of water’s role as a moderator and mediator in protein–substrate interactions, in protein folding and in protein-protein interactions mainly by means of H-bonding formation inside the protein molecule or between molecule and water [46]. 

Over the course of several decades, Dr. Gerald Pollack proved the presence of the “fourth phase of water,” which is formed in bulk water after absorption of radiant energy [19]. It is called Exclusion Zone (EZ) water, which is formed at hydrophilic surfaces, such as biological membranes, most macromolecules, and at phase interfaces. Charge separation takes place in it, since negatively charged hydroxide ions are concentrated along the surface, and positively charged H_3_O^+^ particles are pushed out of the EZ along with particles and microbubbles. Studies have shown that the absorption of UV and the visible light by water leads to the growth of exclusion zones and, in some cases, to its appearance, yet light in the infrared region of the spectrum has been found to be especially effective in impacting EZ water [20]. Because of its property of charge separation, EZ water is suggested to be an energy source for biological processes [47]. Numerous studies in laboratories worldwide show that the interfacial zone differs physically from the bulk zone: it is a more ordered phase of water, appearing to behave like a liquid crystal that can co-exist with the contiguous bulk phase. Negatively charged exclusion zones can be a source of electrons for oxidized molecules, and the low entropy of structured water can equalize the increased entropy of denatured and damaged molecules. Thus, EZs act as universal antioxidants as well. According to G. Pollack, fully built EZs around each protein seem necessary for optimal cellular functioning [48].

Pollack’s model is based on electrostatic laws. Though it allows to explain many experimentally established properties of EZ-water not all its features readily follow from it. On the other hand, long before EZ-water was discovered Emilio Del Giudice and Giuliano Preparata have suggested a model of water where it is represented as a two-phase system in which one fraction of water belongs to the so-called Coherent Domains (CDs), where water molecules oscillate in phase, and the remaining fraction of water constitutes a non-coherent phase. Exclusion zone water has many measured properties of theoretical CDs [49,50]. One of the major features of CDs is that they represent reservoirs of quazi-free electrons that are easily excitable and may be donated to appropriate acceptors. The reason for this lies in the fact that the energy of the excited state of the CD is 12.06 eV, which is very close to the ionization energy of water molecules of 12.6 eV [51]. Ability of EZ-water to be the source of electrons was demonstrated experimentally [48]. The coherent state of water may be significantly stabilized in cases where water is attracted to nearby surfaces, thus shielding its coherent state from the disruptive effects of collisions [52]. Therefore EZ-water has many measured properties of theoretical CDs [49]. They both have notable redox properties, electron exchange, and dynamic stability, which provide for the enhanced ability of the water to stabilize macromolecules present in aqueous systems containing CDs against degradation caused by external factors. As regards humid air irradiated with IR in this study it should be mentioned that in a study on aerosols formed at waterfalls, Madl et al. [53] have shown the presence of negatively charged nanometer-sized and up to 100 nm large water clusters that contain millions of water molecules. Both negative surface charge and the size are in accordance with the properties of CD. Thus, these aerosol water clusters can act as a surface, and exposure to IR is expected to corroborate their coherent properties (that is why we denoted IR-treated water vapor “Coherent Humidity, CoHu”.

In our study we did not treat protein solutions with IR directly, instead we used irradiated humid air as an intermediate for several reasons. Firstly, irradiation of water with IR leads to its heating while microdroplets in the air lose heat quicker. Secondly, water microdroplets have an exclusion zone water layer on their surface [48], and their total surface is much larger than that of liquid water. And finally, water vapor is reported to absorb IR energy more intensely than liquid water [54] making it an inducer of EZ in bulk liquid water. When a coherent IR LED radiation signal is transferred to air with suspended microdroplets, they begin to oscillate coherently and create coherent domains (CDs), which is suggested to be more effective than irradiating liquid water. 

We hypothesize that irradiation of the aquatic environment with light from the infrared region of the spectrum at wavelengths of singlet oxygen phosphorescence leads to formation of exclusion zones and coherent domains in the aqueous media. Further experiments will be conducted to confirm or refute this hypothesis. Thus, singlet oxygen may be a natural source of radiant energy on molecular and cellular levels for building and enhancing EZs. 

## 4. Materials and Methods

### 4.1. Illumination

**Figure 11:**
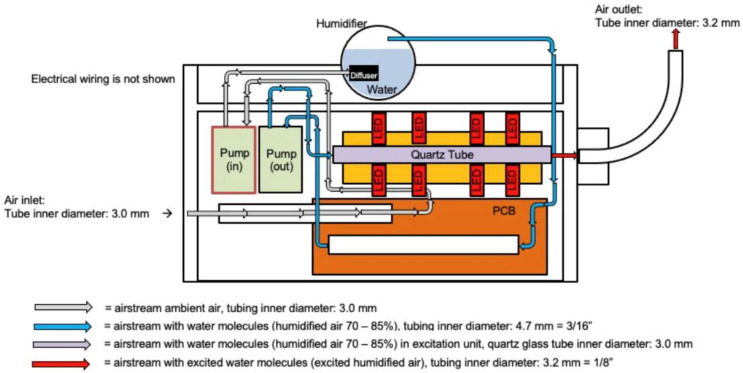
The scheme of the apparatus for CoHu preparation.

CoHu was produced with a NanoVi^®^ device (Eng3 Corporation, Seattle, WA, USA) that humidifies air with water microdroplets 1–10 µm in size using pure distilled water (Figure 11). Humidifier excitation units and a control unit were used. The main feature of the technology is irradiation of the humid airstream with infra-red electromagnetic energy to modify its physico-chemical properties. We have called the modified air humidity coherent humidity (CoHu). This irradiated humid air can be applied for treatment of bulk water or other objects. Unpurified ambient air with initial humidity of 22–26% is used as the carrier gas. Ambient air is pumped (Pump in) and pressed through the diffuser to water in the humidifier glass container. With another pump (out) the humidified air is pressed through the quartz glass tube of the excitation unit equipped with 24 LEDs, a heat sink element and a thermo sensor. Each pump creates an air output of 4 L/min. The humid air (70–85% humidity) goes through a 40 mm long quartz glass tube with an inner diameter of 3 mm. Within glass tube the water vapor absorbs energy that is emitted by the LEDs placed around the tube at a distance of 2.3 mm. The diameter of each LED is 4.7 mm, and they emit IR energy in the range of 1270 nm with a beam cone of 30° and the output power of 700 pW. The control unit allows to switch the LEDs off and to obtain the non-irradiated humid air for control measurements (non-CoHu). Through the 70 cm long flexible plastic outlet with the inner diameter of 3.2 mm CoHu (or non-CoHu) exits the device. 

Enzyme samples were placed in sterile glass flasks of varied volume, depending on the experimental needs, and humid air supply tube was placed 4 cm above the sample surface at ambient light. Several parallel samples were treated with CoHu and non-CoHu for 1 to 5 min depending on experiment design. After treatment samples were used in the experiments within 3 min. Untreated samples were left as controls. 

### 4.2. Horseradish Peroxidase Activity Measurement In Vitro 

Horseradish peroxidase (HRP) type II (RZ > 2.5, 60 U/mg), KH_2_PO_4_, and hydrogen peroxide 30% (*v*/*v*) were obtained from Merck. Potassium-phosphate buffer solution at 0.05 M (pH 6.8) was used as the solvent system for all other reagents and was prepared in deionized water with resistivity of 18.3 MOhm × cm (Millipore, Merck, Darmstadt, Germany).

Specific activity of peroxidase was determined spectrophotometrically (Specord^®^ Plus Analytic Jena spectrophotometer, Germany) in an enzymatic reaction with 2,2-azino(bis(3-ethylbenzothiazoline-6-sulfonicacid)) (ABTS) [27,55,56]. The chromomorphic biocatalytic reaction can easily be monitored by following formation of the ABTS^+•^ radical at the maximum absorption wavelength of 414 nm. One unit (U) is the amount of HRP that catalyzes the oxidation of 1 µmol of ABTS per minute.

The 0.05 M potassium-phosphate buffer was prepared by the following routine. Solution 1: 75 mL of 0.2 M KH_2_PO_4_ was prepared by adding 2.04 g of salt to 75 mL of water; Solution 2: 50 mL of 0.2 M KOH was prepared by adding 0.56 g of salt to 50 mL of water; 33.6 mL of Solution 2 was added to 75 mL of Solution 1, and water was added to obtain 300 mL of the final buffer solution. 

ABTS 0.025 M stock solution was prepared by dissolving 0.0515 g of ABTS in 4 mL of the buffer. Then, 4 µL of the stock solution was added to 2 mL of reaction medium. 

HRP stock solution was prepared by dissolving 0.0125 g of the enzyme in 2 mL (A_403_ = 2.61) of the buffer. Then, the stock solution was diluted 10-fold and 10 µL of this solution was added to 2 mL of the reaction medium.

The reaction medium consisted of 1.9 mL of buffer, 4 µL 30% H_2_0_2_, 4 µL 0.025 M ABTS, and 10 µL HRP. The total reaction lasted 300 s. 

In the course of this test set-up, samples containing intact enzyme in buffer were used as controls; samples containing enzyme treated with non-CoHu were also used for comparison against samples of enzyme treated with CoHu. Also, the impact of treatment of samples with coherent and non-coherent humidity was studied both before and after damaging factors were added.

For 1-min long treatment with CoHu or non-CoHu, 10 µL HRP in 1.9 mL of the buffer were put in 5 mL quartz cuvettes, the air outlet of NanoVi^®^ was placed 4 cm above the solution surface. Control samples were not exposed to the treated airstream.

### 4.3. Alkaline Phosphatase Activity Measurement In Vitro

Materials were purchased from the following sources: Alkaline phosphatase (*E. coli*, M = 190,000), Tris from AppliChem, L-(+)-ascorbic acid, MgCl_2_, CuSO_4_ × 5 H_2_0 from Merck, ZnCl_2_, 2-mercaptoethanol, guanidine HCl from Reachem. Ascorbate stock solution was prepared daily in 50 mM Tris buffer, pH 7.4. All solutions were prepared in 50 mM Tris, pH 9.8, dissolved in water and distilled through a Milli-Q water purification system (Merck, Darmstadt, Germany).

Stock solutions of *E. coli* alkaline phosphatase were prepared by dissolving 200 units and 4 mg of the enzyme in 400 µL of Tris buffer, pH 8.4, containing 50 mM Tris, 1 mM MgCl_2_, 0.1 mM ZnCl_2_. Protein concentration was calculated from the absorbance at 278 nm by using A1% 1 cm = 7.9. 

10 mm quartz spectrophotometer cuvettes with 3 mL of 50 mM Tris buffer were thermostated at 30 °C for 5 min, and a 10 µL aliquot of the enzyme stock solution was added afterward. The reaction was initiated by adding a solution of 4-nitrophenyl phosphate immediately before measuring the optical density at a wavelength of 410 nm (the molar absorption coefficient of 4-nitrophenolate ion is 18,500 M^−1^ × cm^−1^). Measurements were made against thermostated buffer. The enzyme concentration in all experiments was 3 × 10^−^^12^ M, 4-nitrophenyl phosphate—3 mM. 

As with peroxidase, we used controls untreated with treated airstream, as well as controls that were treated with non-CoHu. Five mL quartz cuvettes with alkaline phosphatase in buffer were used for 1-min-long treatment with CoHu or non-CoHu. The air outlet was placed 4 cm above the liquid surface. 

### 4.4. Catalase Activity Measurement In Vitro

The catalase assay was developed by Aebi et al., in 1984 [57]. 

Bovine liver catalase, potassium hydroxide, hydrogen peroxide 30%, KH_2_PO_4_ and Na_2_HPO_4_·2H_2_0 were purchased from Sigma-Aldrich (St. Louis, MO, USA). 

To prepare 50 mM potassium-phosphate buffer with pH 7.0, (a) 6.81 g KH_2_PO_4_ and (b) 8.90 g Na_2_HPO_4_·2H_2_0 were dissolved in distilled water and made up to 1000 mL each. Solutions (a) and (b) were mixed in the proportion 1:1.5 (*v*/*v*). A portion of 0.34 mL 30% hydrogen peroxide was diluted with phosphate buffer to 100 mL.

Bovine liver catalase activity was measured using a UV-Vis spectrophotometer (Specord Analytic Jena, Jena, Germany) using 10 mM H_2_O_2_ as substrate in 50 mM potassium phosphate buffer (pH 7.0). The procession of hydrogen peroxide was followed at 190 nm. 2.9 mL of 10 mM H_2_O_2_ was added to a quartz cuvette, then 0.10 mL of catalase solution containing 300 U/mL was pipetted into the cuvette. The obtained solution was immediately mixed by inversion and the decrease in absorbance was accessed by taking one reading per 1 min for 12 min. Residual quantity of H_2_O_2_ (catalase substrate) in the catalase-assisted degradation reaction was analyzed, which reflects the activity of the enzyme. The higher the hydrogen peroxide concentration in the end of the reaction—the lower the activity of the enzyme. 

Treatment with CoHu or non-CoHu was carried out in 2 mL Eppendorf tubes for 1 min with the airstream outlet 4 cm above the surface of enzyme. 

### 4.5. Heating of Enzymes

Five experimental set-ups were used:

1—samples containing heat-inactivated enzyme in a water bath—inactivated control;

2—samples containing the inactivated enzyme treated with non-CoHu prior to inactivation (Prior/non-CoHu);

3—samples containing the inactivated enzyme treated with CoHu prior to inactivation (Prior/CoHu);

4—samples containing the inactivated enzyme treated with non-CoHu after inactivation (After/non-CoHu);

5—samples containing the inactivated enzyme treated with CoHu after inactivation (Prior/CoHu).

Heating was carried out in quartz cuvettes. After heating, enzyme samples were left at room temperature for 20 min for cooling.

Samples containing 1.9 mL of buffer with 10 µL of horseradish peroxidase were heated at 85 °C for 10 min in a water bath. Samples were then cooled at room temperature for 30 min and divided into test groups and heated control. After cooling, the enzymatic reaction was initiated in every tube by adding H_2_O_2_ and ABTS.

Alkaline phosphatase samples containing 10 µL of enzyme in 3 mL of Tris buffer (pH 8.4) were placed on water bath at 65 °C for 30 min. Then the samples were left at room temperature for 30 min. 

Quartz cuvettes with 0.1 mL of catalase were incubated in 2.9 mL potassium-phosphate buffer at 55 °C for 15 min on a water bath. 

### 4.6. Oxidation with Mixed Function Oxidative System

For tests with oxidative inactivation of enzymes enzyme samples were divided into 5 groups:

1—samples containing oxidized enzyme (Oxidized Controls);

2—samples containing oxidized enzyme treated with non-CoHu prior to oxidation (Prior/non- CoHu);

3—samples containing oxidized enzyme treated with CoHu prior to oxidation (Prior/CoHu);

4—samples containing oxidized enzyme treated with non-CoHu after inactivation (After/non-CoHu);

5—samples containing oxidized enzyme treated with CoHu after inactivation (After/CoHu).

In this experiment enzyme stock solutions were exposed to oxidation and treated with Cohu and non-CoHu before or after oxidation for 3 min. Thus, HRP stock solution was separated in two parts. One was the control, and another was oxidized by the addition of 10 mM ascorbic acid (PanReac AppliChem, Darmstadt, Germany) and 0.1 mM CuCl_2_ (Merck, Darmstadt, Germany). The method of inactivation of enzymes using mixed function solution was performed as previously described by other authors [58,59]. 

Alkaline phosphatase was incubated for 60 min at 35 °C in 50 mM Tris buffer with 10 mM ascorbate in buffer a 10 mM ascorbate with 0.1 mM CuCl_2_. Twenty minutes after samples were removed from the thermostat, aliquots were withdrawn from the mixture and assayed for enzyme activity as previously described [60,61]. Each experiment was repeated 10 times. Each compound was added to the enzyme solution immediately after rapid mixing. The reaction was stopped by the addition of EDTA to 0.5 mM. 

Buffered catalase samples were incubated with 10 mM ascorbate in buffer with 0.1 mM CuCl_2_ for 20 min at 25 °C, removed from the thermostat and used in enzymatic activity measurement 20 min afterwards. 

### 4.7. Denaturation of Enzymes

Stock solutions of enzymes were divided in 6 parts. One of them was used for preparation of control samples that were not denatured or treated with vapor. The second was used for preparation of denatured controls that were not treated with vapor. The third was taken to prepare enzyme samples that were denatured and then treated with non-CoHu for 10 min after unfolding. The fourth was used for preparation of enzyme samples that were treated with non-CoHu for 10 min prior to unfolding. The fifth group of samples was treated with CoHu before unfolding, while the sixth was treated with CoHu after unfolding. The stock solution was 5-times more concentrated than required and was diluted 5-fold either in unfolding or basic buffer depending on the test group it belonged to.

A HRP stock solution was prepared by adding 0.025 g of HRP to 2 mL of potassium-phosphate buffer. Denaturation was performed by diluting this stock solution of the peroxidase (12.5 mg/mL) 5-fold into unfolding buffer (25 mM 2-mercaptoethanol (PanReac, AppliChem, Germany), 3.6 M guanidine HCl, and potassium-phosphate buffer at pH 6.8) and incubating for 12 h at 37 °C in 5 mL plastic tubes. A similar method was described by other authors [62,63].

A highly concentrated alkaline phosphatase stock solution was created by dissolving 4 mg of alkaline phosphatase in 80 µL of Tris buffer, which was then diluted 5-fold in denaturation buffer, containing 25 mM 2-mercaptoethanol, 3.6 M guanidine HCl and Tris buffer, as with peroxidase. The enzyme stock solution was incubated for 10 h with denaturation buffer in 2 mL plastic tubes. Then, 10 µL aliquots of the enzyme were withdrawn for estimation of enzyme activity.

A concentrated catalase solution in buffer with 1500 U/mL was diluted 5-fold in denaturation buffer, containing 25 mM 2-mercaptoethanol, 3.6 M guanidine HCl and potassium-phosphate buffer. The enzyme stock solution was incubated for 10 h with denaturation buffer in 2 mL plastic tubes. Aliquots containing 300 units/mL of the enzyme were withdrawn for estimation of enzyme activity at various time points (30 min, 60 min, 90 min and 120 min) after exposure to unfolding buffer. 

For controls, the enzymes were dissolved and diluted 5-fold in basic buffers, and enzyme activity was accessed after incubation without any other manipulation of the enzymes.

### 4.8. Statistical Analysis

Statistical data processing was performed using a PC with Windows 7 software, Statistica Base and Excel 2013. Results are presented as means with their standard error (Student’s *t*-test).

## Data Availability

Not applicable.
